# Enzymatic and Chemical Syntheses of Vacor Analogs of Nicotinamide Riboside, NMN and NAD

**DOI:** 10.3390/biom11071044

**Published:** 2021-07-16

**Authors:** Lars Jansen Sverkeli, Faisal Hayat, Marie E. Migaud, Mathias Ziegler

**Affiliations:** 1 Department of Biological Sciences, University of Bergen, 5007 Bergen, Norway; lars.sverkeli@uib.no; 2 Department of Biomedicine, University of Bergen, 5009 Bergen, Norway; 3 Mitchell Cancer Institute, University of South Alabama, Mobile, AL 36604, USA; fhayat@health.southalabama.edu (F.H.); mmigaud@southalabama.edu (M.E.M.)

**Keywords:** NAD^+^, vacor, base exchange, Vorbruggen chemistry

## Abstract

It has recently been demonstrated that the rat poison vacor interferes with mammalian NAD metabolism, because it acts as a nicotinamide analog and is converted by enzymes of the NAD salvage pathway. Thereby, vacor is transformed into the NAD analog vacor adenine dinucleotide (VAD), a molecule that causes cell toxicity. Therefore, vacor may potentially be exploited to kill cancer cells. In this study, we have developed efficient enzymatic and chemical procedures to produce vacor analogs of NAD and nicotinamide riboside (NR). VAD was readily generated by a base-exchange reaction, replacing the nicotinamide moiety of NAD by vacor, catalyzed by *Aplysia californica* ADP ribosyl cyclase. Additionally, we present the chemical synthesis of the nucleoside version of vacor, vacor riboside (VR). Similar to the physiological NAD precursor, NR, VR was converted to the corresponding mononucleotide (VMN) by nicotinamide riboside kinases (NRKs). This conversion is quantitative and very efficient. Consequently, phosphorylation of VR by NRKs represents a valuable alternative to produce the vacor analog of NMN, compared to its generation from vacor by nicotinamide phosphoribosyltransferase (NamPT).

## 1. Introduction

**Figure 1:**
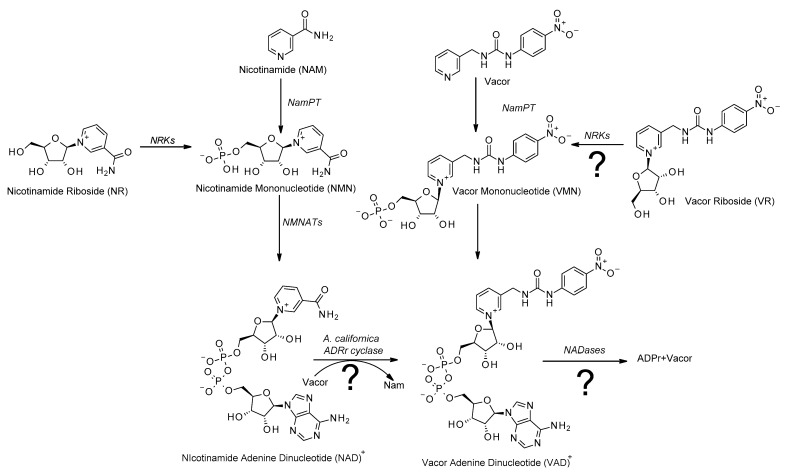
Vacor acts as an analogue to nicotinamide: Overview of the salvage pathway of NAD biosynthesis (left). Nicotinamide is converted to nicotinamide mononucleotide (NMN) in a rate-limiting reaction catalyzed by nicotinamide phosphoribosyl transferase (NamPT). NMN is then converted to NAD in a reaction catalyzed by nicotinamide mononucleotide adenylyl transferases (NMNATs). The alternative NAD precursor nicotinamide riboside (NR) enters the pathway by conversion to NMN catalyzed by nicotinamide riboside kinases (NRKs). Vacor is converted to an NAD analogue through the same pathway, being converted to vacor mononucleotide (VMN) by NamPT and then further converted to vacor adenine dinucleotide (VAD) by NMNAT2. Possible alternative pathways for generating or consuming VAD that are addressed in the present study are indicated with question marks.

Since its discovery in the early 1900s [1], nicotinamide adenine dinucleotide (NAD) has been considered a vital cofactor and signaling molecule for all living organisms. Originally identified as a cofactor for redox reactions [1,2,3], it has since then been shown to serve as a substrate for several important cellular functions, many of which lead to the degradation of NAD [4,5,6,7,8,9]. As NAD is constantly consumed, the cell is dependent on a continuous supply of NAD. In mammalian cells, most NAD is synthesized in a two-step process called the nicotinamide (Nam) salvage pathway (Figure 1). In this process, Nam is converted to nicotinamide mononucleotide (NMN) by nicotinamide phosphoribosyl transferase (NamPT) in a rate-limiting reaction [10]. This reaction requires phosphoribosyl pyrophosphate (PRPP) as co-substrate. NMN is then converted to NAD using ATP in a reaction catalyzed by nicotinamide mononucleotide adenylyl transferases (NMNATs), of which there are three isoforms with different subcellular localization and tissue-specific expression patterns [11]. As Nam is the product of most reactions consuming NAD, the salvage pathway lets the cell recycle Nam as its main NAD precursor. Given the importance of NAD, the ability to control the cell’s NAD pool can be a very valuable asset from a therapeutic perspective [5,12,13,14,15]. As the NAD pool is constantly replenished, one promising strategy for modulating the cell’s NAD levels is interfering with NAD production [15,16,17,18,19,20]. Consequently, the NAD biosynthetic pathways have emerged as a topic of significant research.

One compound that has recently seen a certain resurgence in attention is vacor. Originally developed as a rat poison [21], without a known mechanism of action, recent research has shown that vacor supplementation leads to accumulation of vacor mononucleotide (VMN) and vacor adenine dinucleotide (VAD), that is, vacor-containing analogues of NMN and NAD, respectively [20,22]. This conversion is catalyzed by NamPT and NMNAT2 [20,22], showing that vacor indeed enters the salvage pathway of the NAD biosynthesis (Figure 1). Interestingly, vacor has been shown to act as a potent cytotoxic agent, leading to rapid NAD depletion and subsequent cell death [20,22]. Furthermore, vacor-induced cytotoxicity appears to be selective, affecting primarily neuronal and pancreatic tissue [20,22,23], making vacor an interesting toxin not only from an academic viewpoint, but also as a potential chemotherapeutic agent. The molecular mechanisms of vacor-induced cytotoxicity have remained unknown. However, it is clear that the accumulation of vacor-derived NAD analogs are crucial elements [20,22]. Understanding these mechanisms could identify novel biological processes and eventually new therapeutic targets. Critically, current methods to produce vacor analogs of NAD and its biosynthetic intermediates are rather complex and time consuming, as they involve expression and purification of the enzymes of the salvage pathway of the NAD biosynthesis [20,22]. In this study, we developed an easy, one-step method for synthesizing VAD (Figure 1), exploiting the catalytic properties of commercially available ADP-ribosyl cyclases [24,25].

In addition, while the salvage pathway is the main source of NAD for the cell, alternative NAD precursors can be converted to NAD through different pathways. Nicotinamide riboside (NR), in particular, has been extensively studied recently due to its ability to boost systemic NAD levels when taken as a food supplement [15,18,26,27,28]. NR enters the biosynthetic pathway through nicotinamide riboside kinases (NRKs), which convert NR to NMN in an ATP-dependent reaction [29,30], bypassing the rate-limiting step of the salvage pathway (Figure 1). Here, we present the chemical synthesis of vacor riboside (VR) and demonstrate that it acts as an authentic NR analogue, as it is indeed converted to VMN by NRKs. Thereby, VR could be a useful alternative to vacor, as a tool to study the mechanisms of vacor toxicity. Taken together, we have developed and validated efficient methods to produce vacor analogs of NAD and its biosynthetic intermediates that will be useful to explore the mechanisms of vacor toxicity.

## 2. Materials and Methods

Unless stated otherwise, all reagents were purchased from Sigma-Aldrich (Oslo, NO, USA).

### 2.1. Synthesis of VAD by A. californica ADP-Ribosyl Cyclase

A reaction mix containing 250 µM vacor (greyhound chromatography) and 50 µM NAD^+^ or cADPr in 10 mM Na-phosphate, pH 7.0, was prepared in a total volume of 500 µL. The reaction was started by addition of 0.1 U *A. californica* ADP-ribosyl cyclase (Sigma-Aldrich) and incubated at room temperature for 30 min. The reaction mix was then filtered using a 10 kDa-cutoff spin column (Amicon) at 14,000× *g*, at 4 °C for 10 min, and the flow-through stored at −80 °C for further analysis.

### 2.2. VR Synthesis

In a 50 mL round bottom flask equipped with a stir bar, 3-picolylamine (1.0 eqv, 5 mmol) was dissolved in 50 mL anhydrous chloroform. The round bottom flask was evacuated and filled with argon, then a solution of 4-nitrophenyl isocyanate (1.0 eqv, 5 mmol) in 5 mL anhydrous chloroform was added. The resulting mixture was stirred at RT and the formation of a yellow precipitate was observed immediately. The reaction mixture was stirred at RT overnight, after which time, the yellow precipitate was filtered off, washed with chloroform, and dried under reduced pressure to afford vacor as a yellow powder. Yield 92%, ^1^H NMR (400 MHz; DMSO-*d*_6_) δ, ppm: 9.43 (s, 1H, NH), 8.55 (brs, 1 H, NH), 8.46 (d, *J* = 4.76 Hz, 1 H, Ar-H), 8.14 (d, *J* = 9.32 Hz, 2 H, Ar-H), 7.72 (d, *J* = 7.04 Hz, 1 H, Ar-H), 7.64 (d, *J* = 8.6 Hz, 2 H, Ar-H), 7.37 (q, *J* = 4.8 Hz, 1 H, Ar-H), 7.02 (t, *J* = 5.9 Hz, 1 H, Ar-H), 4.35 (d, *J* = 5.88 Hz, 2 H, CH_2_NH). ^13^C NMR (100 MHz; DMSO-*d*_6_) δ, ppm: 154.98 (CO), 149.19, 148.56, 147.47, 140.98, 135.81, 135.47, 125.53, 123.90, 117.44 (Ar-C), 40.38 (NH-CH_2_); HRMS calcd for C_13_H_13_N_4_O_3_[M + H]^+^ 273.09876 found 273.09971.

To a solution of vacor (1 eqv, 1.8 mmol) in anhydrous CH_3_CN (5 mL), *N,O*-bis(trimethylsilyl)-acetamide (BSA) (2.2 eqv, 4.0 mmol), trimethylsilyl chloride (TMSCl) (1 eqv, 1.8 mmol) and a catalytic amount of ammonium sulfate were added. The resulting mixture was stirred at room temperature for 3 to 4 h under argon, and then concentrated under reduced pressure to afford the crude silylated vacor.

Trimethyl silyl triflate (TMSOTf) (3.9 eqv, 7.3 mmol) was added to a mixture of crude silylated vacor (1 eqv, 1.8 mmol) and D-ribose tetraacetate (1 eqv, 1.8 mmol) in anhydrous 1,2-dichloroethane (15 mL). The reaction mixture was stirred at RT for 3 h. Once the reaction was complete, the mixture was concentrated under reduced pressure and the residue was dissolved in 20 mL reagent-grade methanol. The solution was basified with a saturated aqueous solution of NaHCO_3_ at 0 °C. Once the aqueous methanolic solution reached a pH between 5 and 6, the addition of NaHCO_3_ was stopped and the resulting mixture was concentrated on the rotatory evaporator with no heating. The crude product was washed with diethyl ether 2–3 times and dried under reduced pressure to afford VR triacetate in a solid form. Yield 68%, ^1^H NMR (400 MHz; MeOD) δ, ppm: 9.03 (s, 1 H, Ar-H), 8.96 (d, *J* = 6.2 Hz, 1 H, Ar-H), 8.58 (d, *J* = 8.0 Hz, 1 H, Ar-H), 8.09 (t, *J* = 6.7 Hz, 1 H, Ar-H), 8.04 (d, *J* = 7.34 Hz, 2 H, Ar-H), 7.51 (d, *J* = 8.47 Hz, 2 H, Ar-H), 6.41 (d, *J* = 4.24 Hz, 1 H, H-1), 5.42 (t, *J* =4.94 Hz, 1 H, H-2), 5.32 (t, *J* = 5.24 Hz, 1 H, H-3), 4.68 (q, *J* = 3.85 Hz, 1 H, H-4), 4.55 (d, *J* = 3.16 Hz, 2 H, CH_2_NH), 4.50 and 4.36 (AB part of ABX system, 2 H, J_AB_ = 12.8 Hz, J_AX_ = 4.0 Hz, J_BX_ = 2.7 Hz, H5′), 2.03 (s, 6 H, Ac), 2.00 (s, 3 H, Ac). ^13^C NMR (100 MHz; MeOD) δ, ppm: 170.51 (CO), 169.97 (CO), 169.75 (CO), 155.71 (NHCONH), 146.57, 145.96, 142.14, 141.93, 139.64, 139.19, 127.86, 124.46, 117.76 (Ar-C), 97.39 (C-1), 83.05 (C-4), 76.20 (C-2), 69.09 (C-3), 62.36 (C-5), 40.57 (CH_2_NH), 20.63 (Me), 19.32 (Me), 18.87 (Me).

The crude vacor riboside triacetate (1 eqv, 1.8 mmol) was dissolved in 15 mL anhydrous methanol. After 15 min of stirring at RT, 10 mL of an NH_3_ solution (0.5N) in 1,4-dioxane was added and the resulting mixture was stirred at RT overnight. The reaction progress was monitored by TLC. Upon completion of the reaction, the solvent was removed under reduced pressure and the crude product was purified by flash column silica chromatography through gradient elution by using hexane: ethylacetate (1:1), ethylacetate: MeOH (1:1) and water: MeOH (1:1) as eluents. Yield 62%, ^1^H NMR (400 MHz; D_2_O) δ, ppm: 9.00 (s, 1 H, Ar-H), 8.91 (d, *J* = 6.12 Hz, 1 H, Ar-H), 8.45 (d, *J* = 7.92 Hz, 1 H, Ar-H), 8.34 (s, 1 H, Ar-H), 8.04–7.99 (m, 2 H, Ar-H), 7.33 (d, *J* = 9.2 Hz, 1 H, Ar-H), 6.06 (d, *J* = 4.6 Hz, 1 H, H-1), 4.55 (s, 2 H, CH_2_NH), 4.38–4.35 (m, 1 H, H-3), 4.22 (t, *J* = 4.52 Hz, 1 H, H-2), 3.88–3.76 (AB part of ABX system, 2 H, J_AB_ = 12.9 Hz, J_AX_ = 2.92 Hz, J_BX_ = 3.8 Hz, H5′). ^13^C NMR (100 MHz; D_2_O) δ, ppm: 170.83 (Ar-C), 156.62 (CO), 145.81, 145.29, 141.84, 141.22, 139.08, 138.42, 127.92, 125.17, 118.25 (Ar-C), 99.52 (C-1), 87.59 (C-4), 77.50 (C-2), 69.98 (C-3), 60.53 (C-5), 40.59 (CH_2_NH); HRMS calcd for C_18_H_21_N_4_O_7_[M + H]^+^ 405.14102 found 405.14214.

### 2.3. Cultivation, Vacor/VR Treatment and Nucleotide Extraction of 293 Cells

293 cells were cultivated in Dulbecco’s modified Eagle’s medium (Gibco) supplemented with 10% (*v*/*v*) fetal bovine serum (FBS), 2 mM glutamine and penicillin (100 U/mL)/streptomycin (100 µg/mL) (normal medium). The cells were cultured at 37 °C in a humidified atmosphere of 5% CO_2_. Prior to vacor/VR treatment, the cells were seeded in 12-well plates (200,000 cells/well) in 1 mL normal medium. The day after seeding, the cells were washed once with PBS and the medium was changed to the normal medium supplemented with 100 µM vacor, 100 µM VR or no supplement (control). The cells were then incubated for 24 h before extraction.

For nucleotide extraction, the cells were placed on ice, the growth medium was removed, and the cells were washed twice with 0.5 mL ice-cold PBS. Then, 0.5 mL ice-cold 80% (*v*/*v*) LC-MS-grade methanol was added to each well and the cells were incubated at 4 °C on a shaker for 20 min. After incubation, the cells were detached from the wells using a cell scraper and the samples were transferred to 1.5 mL tubes. To ensure full transfer, the wells were washed with 0.3 mL of ice-cold 80% (*v*/*v*) LC-MS-grade methanol. The samples were subsequently frozen at −80 °C. On the day of LC-MS analysis, the samples were thawed on a rotating wheel at 4 °C before centrifugation at 16,000× *g* for 20 min at 4 °C. After centrifugation, 0.6 mL of the supernatant was transferred to a new tube and 0.6 mL acetonitrile was added to each sample.

### 2.4. LC-MS Analysis

Separation of the nucleotides by liquid chromatography was done using a SeQuant ZIC-cHILC column (100 × 2.1 mm, 3 µm; Merck) in a Dionex UltiMate 3000 liquid chromatographer coupled to a QExactive mass spectrometer (Thermo Scientific, Oslo, NO, USA). The column compartment was kept at 30 °C during the run. The injection volume for all samples was 10 μL and the flow rate was kept at 0.3 mL/min.

The mobile phase consisted of 20 mM ammonium acetate, pH 6.8 (Buffer A), and acetonitrile (Buffer B). The gradient was set as follows: Runs were started with 80% Buffer B for 1 min (flowthrough). The concentration of Buffer B was further decreased to 70% over 11 min for separation before being brought to 5% over 1 min for washout. After 3.5 min at 5% Buffer B, the concentration of Buffer B was returned to 80% over 1 min for equilibration. The column was equilibrated at 80% B for 3.5 min before the next run. Electrospray ionization was performed using the positive ion polarity mode and a spray voltage of 3.5 kV. The sheath flow gas flow rate was 48 units, with an auxiliary gas flow rate of 11 units and a sweep gas flow rate of 2 units.

Mass spectra were recorded using targeted single-ion monitoring at positive polarity, at a resolution of 70,000. Fragmentation spectra were recorded using MS/MS in parallel reaction monitoring at a resolution of 70,000, with the normalized collision energy set to 18 units. Data analysis was conducted in the Thermo Xcalibur Qual Browser (Thermo Scientific, Oslo, NO, USA).

### 2.5. Recombinant Expression and Purification of NRKs

Pellets from bacterial expression cultures were resuspended in 10 mL resuspension buffer (20 mM Na-PO4 pH 8.0, 300 mM NaCl) and lysed by 6 × 10 s sonication on ice. The cell lysate was then centrifuged for 20 min at 13,000 rpm. During this centrifugation, 1 mL Ni-NTA matrix (Qiagen) was transferred to a 15 mL tube and equilibrated with resuspension buffer by 5 min incubation with 10 mL buffer on a rotating wheel, followed by 3 min centrifugation at 2000× *g*, repeated twice. After centrifugation of the cell lysate, the supernatant was added to the Ni-NTA matrix and was incubated for 1 h at 4 °C while rotating to allow binding of the His-tagged protein to the Ni-NTA matrix. After incubation, the tube was centrifuged for 2 min at 2000× *g*. The supernatant was removed, and the matrix was washed by incubation with 10 mL washing buffer (20 mM Na-PO4 pH 8.0, 300 mM NaCl, 20 mM Imidazole) for 5 min while rotating, followed by 2 min centrifugation at 2000× *g*, repeated twice. After washing, the matrix was resuspended in 2 mL washing buffer and transferred to a column equilibrated with washing buffer. The Ni-NTA matrix was allowed to pack on top of the column by gravity. Elution was performed in three fractions by the addition of firstly 300 μL, and then twice 500 μL of the elution buffer (20 mM Na-PO4 pH 8.0, 300 mM NaCl, 500 mM Imidazole). To assess the stepwise purification of the protein, samples were collected of the pellet and supernatant after lysis, after binding to the column and after washing, and analyzed using SDS-PAGE.

### 2.6. Synthesis of NMN/VMN by NRKs

A reaction mix containing 100 µM VR or NR with 500 µM ATP in 10 mM Na-phosphate, pH 7.0, was prepared in a total volume of 500 µL. The reaction was started by addition of 5 µg NRK1/NRK2 and incubated at room temperature for 60 min. The reaction mix was then filtered using a 10 kDa-cutoff spin column (amicon) at 14,000× *g*, at 4 °C for 10 min, and the flow-through stored at −80 °C for further analysis.

### 2.7. Cell Proliferation Assay

293 cells were seeded, 10,000 cells/well, in a 96-well plate in a high-glucose DMEM medium (Gibco) supplemented with 10% FBS. At 24 h after seeding, the medium was exchanged with a medium containing 100 µM vacor, 100 µM VR or no supplement (control). The cells were then transferred to an IncuCyte Zoom live cell imager. Growth rate was assessed by monitoring cell confluency using phase-contrast microscopy at 2 h time intervals for 48 h.

### 2.8. Cleavage of VAD/NAD Using A. fumigatus NADase

The reaction mix was prepared in 500 µL reaction buffer containing 50 mM Tris-HCl, pH 8.0, 500 µM CaCl_2_ and 150 mM NaCl, supplemented with 10 µM VAD/NAD. The reaction was started by the addition of 10 ng *A. fumigatus* NADase [31] and incubated at room temperature for 30 min. The reactions were then filtered using a 10 kDa-cutoff spin column at 14,000× *g*, at 4 °C for 10 min, and the flow-through stored at −80 °C for further analysis.

## 3. Results

Vacor is commercially available and can therefore be used as a starting material to produce NAD analogs. As it will be described below, while VAD can be generated in a single enzymatic step, VR needs to be chemically synthesized.

### 3.1. Single-Step Generation of VAD Using Aplysia californica ADP Ribosyl Cyclase

The enzymatic synthesis of VAD described so far requires at least two enzymes that need to be recombinantly produced. Moreover, this two-step enzymatic synthesis requires the co-substrates PRPP and ATP (Figure 1). We reasoned that the well-known base-exchange reaction [24,25,32], which is an exceptional feature of ADP-ribosyl cyclases, could be exploited for a simplified route to produce VAD. Among others, these cyclases mediate the substitution of the nicotinamide moiety in NAD by nicotinic acid (the other vitamin B3 form of the pyridine base), thereby generating NAAD, a physiological intermediate of the Preiss–Handler NAD biosynthetic pathway [33].

**Figure 2:**
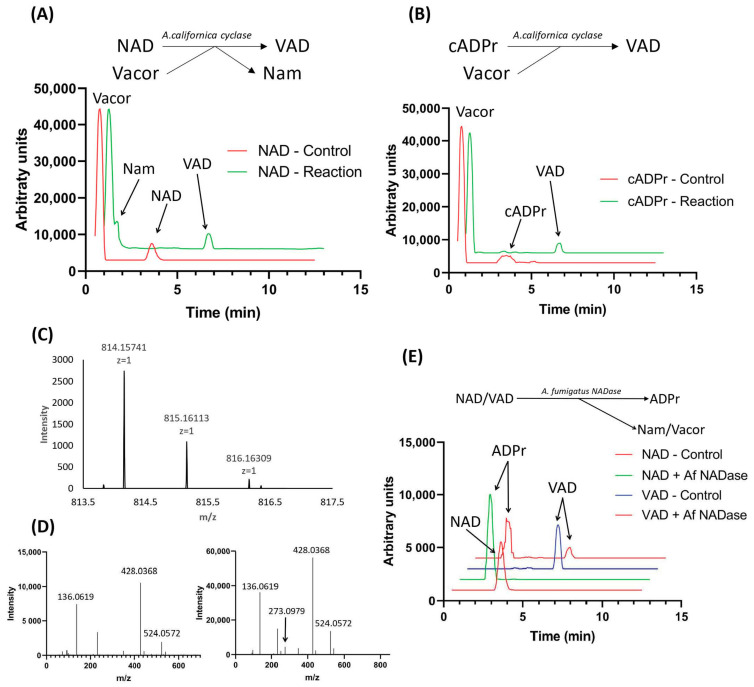
Synthesis of VAD by *Aplysia californica* ADP-ribosyl cyclase through base exchange. (**A**) Scheme of VAD production utilizing *A. californica* ADPr-cyclase activity with vacor and NAD as substrates. Chromatograms show the reaction and control using 50 µM NAD^+^ with 5-fold excess of vacor followed by 10 min incubation using 0.1 U enzyme, quantified by LC-MS. (**B**) Scheme of VAD production utilizing *A. californica* ADPr-cyclase activity with vacor and cADPr as substrates. Chromatograms show the reaction and control using 50 µM NAD^+^ with 5× excess of vacor followed by 10 min incubation using 0.1 U enzyme, detected by LC-MS. (**C**) MS spectrum of VAD produced using the *A. californica* ADPr-cyclase-catalyzed reaction shown in 2B. (**D**) Fragmentation spectra of NAD control (left) and VAD (right) produced using the *A. californica* ADPr-cyclase-catalyzed reaction shown in 2B. (**E**) Cleavage of NAD and VAD by *A. fumigatus* NADase. Chromatograms show the reactions and controls of 10 µM substrate incubated for 30 min using 10 ng enzyme, detected by LC-MS.

We tested this possibility using commercially available *Aplysia californica* ADP-ribosyl cyclase in a one-step reaction with vacor and NAD as substrates (Figure 2A, top). The reaction mix was set up as described in Materials and Methods with a 5× excess of vacor to NAD. Following incubation for 10 min, the reaction mix was analyzed by LC-MS (Figure 2A bottom), showing that NAD was completely converted to VAD. This reaction yields Nam as a side product as the base on NAD is exchanged with vacor. While the base exchange activity of *Aplysia californica* ADP-ribosyl cyclase was proven efficient in producing VAD, the same enzyme is also capable of catalyzing the reverse cyclase reaction, adding a base onto cyclic ADP-ribose (cADPr) [24]. We performed this reaction in a similar manner to the base-exchange reaction, using cADPr as substrate in the presence of a 5-fold excess of vacor. This reaction yielded a complete conversion of cADPr to VAD, with no side product produced (Figure 2B).

The ms spectrum of the product formed matched the expected *m*/*z* and natural isotope distribution of VAD (Figure 2C). To further confirm the identity of the reaction product, we subjected the product to fragmentation analysis using LC-MS-MS, using NAD^+^ as control. The fragmentation spectrum for the reaction product (Figure 2D right) is highly similar to that of NAD (Figure 2D left), with both spectra containing major fragments at *m*/*z* = 136 and *m*/*z* = 428, indicating the presence of an adenosine moiety and an ADP-moiety. Additionally, the reaction product has a fragment at *m*/*z* = 272, corresponding to the expected mass of vacor. Taken together, this demonstrates that the reaction product has incorporated vacor as part of its structure, confirming that it is indeed VAD.

Having successfully produced VAD, we wanted to investigate whether VAD can serve as a substrate for NAD-consuming enzymes. To do this, we used the newly discovered fungal NADase from *Aspergillus fumigatus* [31]—an enzyme shown to possess pure NADase activity, cleaving NAD into ADPr and Nam—in a reaction containing our synthesized VAD, using NAD as a control. The reaction was analyzed using LC-MS (Figure 2E), revealing that the NADase is capable of using both VAD and NAD as substrate, yielding ADP-ribose and the corresponding base as products.

### 3.2. VR Is Converted to VMN by NRKs

**Figure 3:**
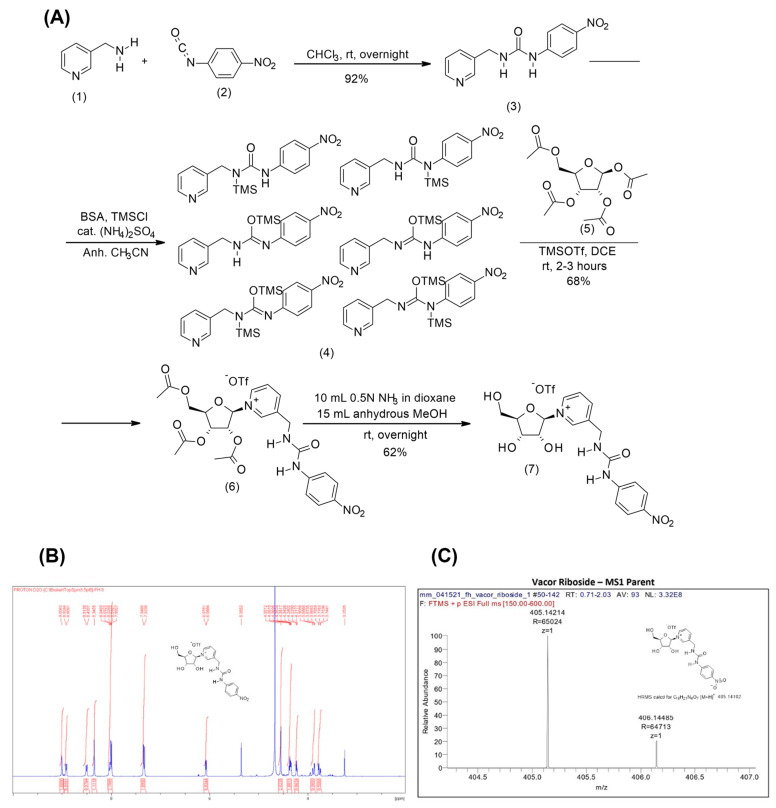
Chemical synthesis of VR. (**A**) Scheme of the synthesis of VR from vacor via Vorbruggen chemistry. (**B**) NMR spectrum of the synthesized VR. (**C**) Mass spectrum of VR.

Next, we wanted to investigate whether vacor derivatives can be generated through alternative entry points in the NAD biosynthetic pathway. In order to do this, we synthesized a vacor analogue of the alternative NAD precursor NR, VR, as described in Materials and Methods. The synthesis of vacor was performed according to the literature [34] and its identity confirmed by NMR and MS spectrometry. The ribosylated derivative of vacor was obtained via Vorbruggen chemistry (Figure 3A) for which vacor was silylated using *N,O*-bis(trimethylsilyl)acetamide and trimethylsilyl chloride with a catalytic amount of ammonium sulfate. Any other silylation methods proved mediocre and led to poor outcomes in the generation of the triacetylated nucleoside. Crucially, the ribosylation of the silylated vacor occurred with complete stereoselectivity and in reproducible good yields using trimethylsilyltriflate as a Lewis acid. The beta-stereochemistry at the C1′ position of the nucleosidic linkage was confirmed by the chemical shift and the multiplicity of the hydrogen at the C1′ position (6.41, d, *J* = 4.24 Hz, 1 H, H-1), which matches that observed in nicotinamide riboside triacetate [35]. The subsequent removal of the three acetyl groups was achieved under mild reaction conditions that, in addition to the saponification reaction, also led to the partial hydrolysis of the glycolytic bond and release of vacor. The crude mixture was therefore purified by silica chromatography. Overall, the fully deprotected VR was obtained at a 39% yield from 3-picolylamine. The identity and purity of the final VR product was confirmed using NMR and mass spectroscopy (Figure 3B,C).

**Figure 4:**
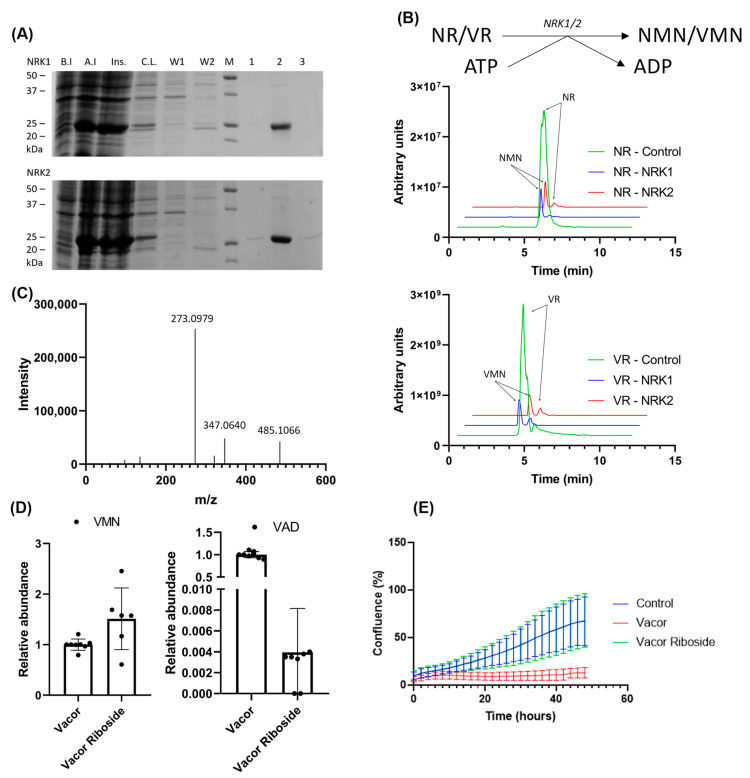
VR is converted to the NMN analogue, VMN, by both NRK 1 and 2. (**A**) Purification of NRK1/2 using immobilized metal ion chromatography, analyzed by SDS-PAGE. B.I.—before induction; A.I.—after induction; Ins.—insoluble fraction; C.L.—clear lysate; W1—wash 1; W2—wash 2; M—marker; 1—elution fraction 1; 2—elution fraction 2; 3—elution fraction 2. (**B**) Scheme of VAD/NAD production utilizing the NRK activity with VR/NR and ATP as substrates. Chromatograms show the reaction (VR) and control (NR) using 100 µM VR or NR, respectively, with 5-fold excess of ATP followed by 60 min incubation using 10 µg enzyme, analyzed by LC-MS. (**C**) Fragmentation spectrum of VMN synthesized from VR by NRKs. (**D**) Abundance of VMN (left) and VAD (right) in 293 cells after 24 h treatment with 100 µM vacor or VR. Metabolites were extracted in methanol and analyzed using LC-MS. All values normalized to the average concentration found in cells treated with vacor. (**E**) Growth curve of the 293 cells in high-glucose DMEM supplemented with 100 µM vacor, 100 µM VR or control (no supplement). The graph shows the confluence measured for 48 h after supplementation, using IncuCyte Zoom live cell imaging.

In NAD biosynthesis, NRK1 and 2 phosphorylate NR to NMN. To test whether NRKs are also capable of phosphorylating VR to VMN, we overexpressed both isoforms in *E. coli* and purified the enzymes using immobilized metal ion chromatography (Figure 4A). An enzymatic reaction using the purified NRKs and our synthesized VR was performed as described in Materials and Methods, with a 5× excess of ATP. The reaction products were analyzed by LC-MS (Figure 4B), showing that both NRK1 and NRK2 were able to completely convert VR to VMN. It should be noted that due to the inclusion of a negatively charged phosphate, the nucleotide products are less efficiently ionized than the nucleoside substrates. This, in turn, means that the reaction products in this case yield a far lower signal compared to the substrates. To verify the identity of the reaction product, we performed a fragmentation analysis using LC-MS-MS (Figure 4C). The major fragmentation ion present is at *m*/*z* = 273, which is the expected *m*/*z* of a vacor moiety. Additionally, a major fragment is observed at *m*/*z* 485, corresponding to the expected mass of the full VMN molecule, indicating that the product formed is indeed VMN.

Having shown that VR can be converted to a product by enzymes of the NAD biosynthetic pathway, we next treated the 293 cells, which are known to express both NRK and NamPT activity, with 100 µM VR or vacor for 24 h. Following extraction in ice-cold methanol, the presence or absence of vacor products in the samples was analyzed using LC-MS (Figure 4D). Interestingly, we observed an accumulation of VMN in cells treated both with VR and vacor, indicating that NRKs are able to convert VR to VMN also in a cellular system.

However, VAD—the downstream product generated by NMNAT2—was only observed when the cells were treated with vacor. Following up on this, we wanted to investigate whether this difference in accumulation of vacor products affected the toxic effect of the treatment. We therefore performed a growth curve experiment over 48 h using 293 cells treated with 100 µM vacor or VR. The cells were monitored using IncuCyte, and cell confluency was determined every 2 h (Figure 4E). As expected, cells treated with vacor showed impaired growth and cell death, with a confluency of 27% compared to the control at 24 h, decreasing to 19% at 48 h. Cells treated with VR exhibited no signs of growth impairment compared to the control cells.

## 4. Discussion

As vacor has gained attention due to its potential to enter the NAD biosynthetic pathway, with downstream vacor products acting as toxins, a potential use case for vacor products has emerged. In this paper, we demonstrate an alternative method for producing VAD, using *Aplysia californica* ADP ribosyl cyclase in a one-step reaction using only commercially available materials. Compared to the method previously used to produce VAD [20,22], the approach presented here is cost-effective and less complex (fewer enzymes, substrates and products), making the final purification of VAD also easier.

Further, we show that not only vacor, but a vacor analogue of the alternative NAD precursor NR, can be converted into a vacor product by NRKs, indicating that VR is also converted in the NAD biosynthetic pathway. The phosphorylation of VR to VMN was confirmed in cell culture experiments, demonstrating that vacor analogues of NAD precursors can enter the NAD biosynthetic pathway from several different entry points. While both NamPT and NRKs are ubiquitously expressed, expression levels across tissues vary. While Vacor has been shown to mainly affect neuronal and pancreatic cells [20], different Vacor analogues might exhibit different cytotoxic profiles. In particular, the fact that VR conversion to VMN is not dependent on NamPT activity can be important. NamPT is the rate-limiting enzyme of the salvage pathway [10] and a major contributor in maintaining cellular NAD levels. NamPT is also subject to negative feedback inhibition by NAD [36], unlike the NRKs. As both NRKs are capable of synthesizing VMN, using VR as an entry point for vacor toxification could potentially prove efficient in a wide array of cell lines and tissues.

In this context, it is very interesting to observe that both vacor and VR cause accumulation of VMN in 293 cells. However, accumulation of VAD, with subsequent cell death, was only observed in the vacor-treated cells, indicating that accumulation of VAD is necessary for vacor-induced cell death. Interestingly, we observed that VAD can act as a substrate for NAD-consuming enzymes, as both *A. californica* ADP-ribosyl cyclase and *A. fumigatus* NADase accept it as a substrate. This raises the question of whether vacor/VR or their products might enter other biological pathways, opening up the possibility that VMN/VAD might be further metabolized in the cell. As the exact mechanisms behind vacor-induced cytotoxicity are not well understood, with different explanations proposed [20,22], this is a highly interesting topic that warrants further investigation.

## Data Availability

All relevant data of this study are presented. Additional data will be provided upon request.
